# Quantitative systems toxicology approach that integrates PBPK and core hepatic metabolism: a case study with valproic acid

**DOI:** 10.3389/fphar.2026.1768190

**Published:** 2026-03-20

**Authors:** Vipul Gupta, Heeseung Jo, Ciarán P. Fisher, Andrzej M. Kierzek

**Affiliations:** 1 Quantitative Systems Toxicology and Safety, Certara Predictive Technologies, Sheffield, United Kingdom; 2 Non-Clinical Safety, GlaxoSmithKline Research and Development Ltd., Stevenage, United Kingdom; 3 Applied BioSimulation, Certara Predictive Technologies, Sheffield, United Kingdom; 4 School of Biosciences, University of Surrey, Guildford, United Kingdom

**Keywords:** hepatic steatosis, new approach methodologies, PPARα, quantitative systems toxicology, valproic acid

## Abstract

New approach methodologies (NAMs) are advancing the reduction of animal testing by promoting human-relevant safety assessments across diverse applications, including cosmetics, pharmaceuticals, and environmental chemicals. Among these methodologies, computational models like quantitative systems toxicology (QST) have emerged as powerful tools, enabling the simulation of mechanisms underlying drug (or chemical) induced toxicity to predict potential adverse outcomes. Valproic acid (VPA), a treatment for epilepsy, convulsions and bipolar disorder, is associated with a risk of drug-induced liver injury. The mechanism of hepatotoxicity is not fully understood, although VPA’s competitive inhibition of fatty acid metabolism via carnitine palmitoyl transferase 1 (CPT1) in the liver is a leading hypothesis. In this study, we employed a QST approach by integrating a physiologically-based pharmacokinetic model of VPA with the large-scale liver metabolism model HEPATOKIN1 to evaluate whether simulated VPA dosing with increasing CPT1 inhibition predicts clinically relevant markers of lipid metabolism disruption and hepatic triacylgyceride (TAG) accumulation, an early maker for hepatic steatosis. The integrated model predicted a dose-dependent increase in hepatic TAG as a result of the competitive inhibition of CPT1 by VPA, qualitatively consistent with clinical observations. Notably, explicit inclusion of PPARα-mediated regulatory effects on key liver enzymes was essential to ensure biologically consistent outcomes in liver metabolism. These results highlight the necessity of including relevant regulatory pathways in QST applications to achieve credible and physiologically relevant safety predictions.

## 1 Introduction

Valproic acid (VPA) is a wide-ranging therapeutic agent primarily utilized in the treatment of epilepsy and convulsions, but also used to treat migraine, schizoaffective disorder, and bipolar disorder for its mood-stabilising properties (Tomson et al., 2016; Lagace et al., 2004). A significant clinical concern associated with VPA is its potential to cause liver damage; however, the exact mechanisms underlying VPA-induced hepatotoxicity remains poorly understood (Ezhilarasan and Mani, 2022; Chang and Abbott, 2006). Among the various hypotheses, one prominent theory suggests that, as a branched, short-chain fatty acid, VPA competitively inhibits catabolism of fatty-acids through the β-oxidation pathway, an essential process in fatty acid metabolism (Silva et al., 2008). Reduced fatty-acid catabolism through this pathway can result in metabolic-dysfunction and drug-induced liver injury (DILI). Clinically, this can present as alterations in metabolic markers - plasma triglycerides (TAG), lipoproteins, and total serum cholesterol - and associated hepatic steatosis, potentially progressing to elevated liver injury markers such as aspartate aminotransferase (AST), alanine aminotransferase (ALT), and bilirubin (Nanau and Neuman, 2013; Ahangar et al., 2017).

The quantitative systems toxicology (QST) approach aims to quantitatively understand the adverse effects of chemicals on a living organism, from molecular alterations to phenotypical observations, through integrating computational modelling and simulation, and experimental methods (Beattie et al., 2024; Goldring et al., 2025). More established approaches under the systems biology umbrella, such as quantitative systems pharmacology (QSP) and physiologically-based pharmacokinetic (PBPK) modelling apply the same computational tools to mechanistically understand pharmacodynamics and pharmacokinetics, respectively (Jones and Rowland-Yeo, 2013). The evermore routine application of these approaches to inform decision making in the drug development pipeline and their increasing acceptance by regulatory authorities (Tan et al., 2018; El-Khateeb et al., 2021) is reflective of an industry wide adoption of model-informed drug discovery and development (MID3).

Several mathematical models of varying scale and mechanistic resolution have been developed to simulate liver function and chemical-induced liver injury. Notable examples include DILIsym® and HEPATOKIN1 (Berndt et al., 2018) among numerous others (Siler, 2022; Schleicher and Dahmen, 2018; Dutta-Moscato et al., 2014; Ashworth et al., 2016). DILIsym® is a multi-scale mechanistic model that integrates cellular toxicity with systemic liver injury biomarkers such as ALT, AST, and bilirubin, and has been validated against exemplar compounds known to cause DILI (Eichenbaum et al., 2020), including VPA-induced liver injury via mitochondrial dysfunction, bile acid transport inhibition, and production of reactive oxygen species; however, it does not specifically model hepatic steatosis (Lakhani et al., 2023). Expanding on this framework, NAFLDsym® (Siler, 2022) incorporates fatty acid dynamics, inflammatory cells, and hepatic stellate function to simulate metabolic dysfunction-associated fatty liver disease (MAFLD, formerly non-alcoholic fatty liver disease) and metabolic dysfunction-associated steatohepatitis (MASH, formerly non-alcoholic steatohepatitis) (Kanwal et al., 2024). This expansion has enabled the prediction of treatment efficacy for both MAFLD and MASH. In contrast, the HEPATOKIN1 (Berndt et al., 2018) model focuses on modelling hepatocyte functions at the molecular and cellular scale, enabling simulation of cell-level processes that contribute to DILI. HepatoNet1 (Gille et al., 2010), originally developed as a metabolic network model, was subsequently expanded into HEPATOKIN1, resulting in a highly detailed kinetic model of hepatocyte metabolism at the genome scale, comprehensively representing metabolic pathways for glycogen, fructose, fatty acids, ketones, and other substrates. This genome-scale resolution enables the incorporation of specific cellular-level mechanisms of DILI in relation to these metabolic pathways and facilitates the simultaneous tracking of numerous metabolic substrates, offering insights not present in the multi-scale models DILIsym® or NAFLDsym®.

It is vital to recognize model limitations to ensure appropriate use in DILI assessment. A notable limitation among the models discussed, including HEPATOKIN1 (Berndt et al., 2018), is the absence of relevant adaptive feedback mechanisms, which are fundamental to the non-linear behaviour of biological systems and the regulation of processes such as inflammation (Qian et al., 2015), differentiation (Laudadio et al., 2012), and metabolism (Barbier et al., 2003; Kersten and Stienstra, 2017). The transcription factor peroxisome proliferator-activated receptor α (PPARα) plays a vital role in lipid metabolism by regulating the expression of several enzymes essential for hepatocyte metabolism. PPARα is particularly integral in modulating the β-oxidation of fatty acids, acting as a critical regulatory node in lipid homeostasis (Dreyer et al., 1993; Minnich et al., 2001). Its influence extends to the regulation of gluconeogenesis and amino acid metabolism and may be especially important when simulating the effects of chemical-induced disruption of hepatic fatty acid metabolism (Todisco et al., 2022; Kersten, 2014; Gutgesell et al., 2009; Li et al., 2014; Maldonado et al., 2018). Previous systems modelling work used a quasi-steady state approach to simulate dynamic regulation of hepatic metabolism; using the HepatoNet1 (Gille et al., 2010) model, showed that increased flux towards intracellular TAG in response to fatty-acid treatment was dependent on PPARα-mediated regulation (Maldonado et al., 2018).

In this study, we develop a multi-scale QST model integrating the cellular-level liver metabolic model, HEPATOKIN1, with a human PBPK model of VPA to simulate VPA-induced disruption of the β-oxidation pathway and predict hepatotoxic potential. The presence of non-linear plasma concentrations of VPA with increasing dose, driven by concentration-dependent plasma protein binding (FDA, 2016; Zaccara et al., 1988), underscores the importance of PBPK to accurately predict VPA concentrations in the liver. This approach includes a semi-quantitative evaluation of PPARα′s regulatory effects, serving as a case study to demonstrate the role of regulatory feedback mechanisms as an essential consideration for application of QST models.

## 2 Methods

### 2.1 Minimal physiologically based pharmacokinetic model for VPA

**FIGURE 1:**
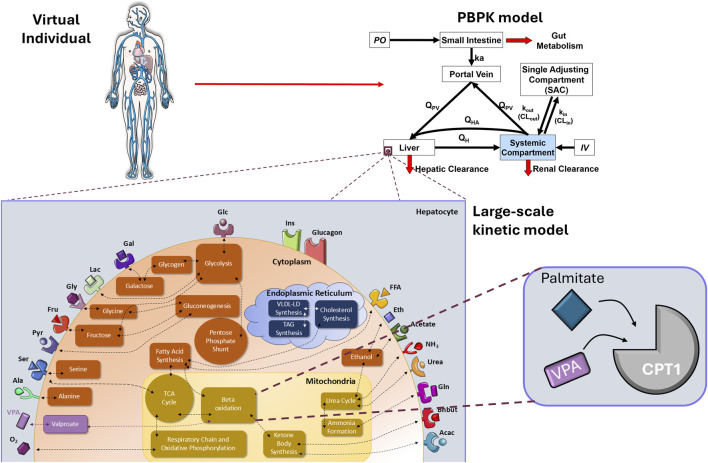
Schematic overview of the integrated QST model. A single population representative virtual individual is used to describe physiological parameters of the human minimal PBPK model of VPA to simulate each VPA dosing scenario. The resulting simulated systemic free VPA concentrations are then linked to hepatocyte metabolism within the large-scale kinetic model, where VPA (purple rectangle) acts as a competitive inhibitor for CPT1-driven β-oxidation of the fatty acid palmitate (blue diamond). In this large-scale kinetic model, each large block represents a different metabolic pathway, and each colour corresponds to a distinct cellular compartment. CPT1, carnitine palmitoyltransferase 1; IV, intravenous administration; k_a_, absorption rate; k_in_ (CL_in_)/k_out_ (CL_out_), in and out, respectively, exchange rate between systemic compartment and single adjusting compartment; PBPK, physiologically based pharmacokinetic; PO, oral administration; Q_PV_, blood flow rate of portal vein; Q_HA_, blood flow rate of hepatic artery; QST, quantitative systems toxicology.

We developed a minimal PBPK (mPBPK) model using the Simcyp Simulator (v23; Certara Inc., Simcyp Division Sheffield, UK) to predict the free plasma concentration of VPA in humans, as previously published in an Organisation for Economic Co-operation and Development (OECD) case study (Escher et al., 2020), with parameter values detailed in Supplementary Table S1. PBPK models simulate drug absorption, distribution, metabolism, and elimination (ADME) using physiological compartments representing organs like blood, liver, and intestines, calibrated by physiological parameters such as body weight, liver weight, and blood volume. The mPBPK approach uses minimal compartmentation to adequately model drug pharmacokinetics (Jones and Rowland-Yeo, 2013) (Figure 1), typically including gut absorption, liver metabolism, and renal clearance. The details of the PBPK simulator and Simcyp’s quality assurance system are provided elsewhere (Ke et al., 2016; Rowland Yeo et al., 2024; Ezuruike et al., 2022). The VPA PBPK model was parameterised using an in vitro-to-in vivo extrapolation (IVIVE) approach, and its predictive performance was verified against published clinical data (Georgoff et al., 2018). VPA exhibits concentration-dependent protein binding (FDA, 2016), which is included into the mPBPK model by accounting for its binding to plasma proteins, specially human serum albumin (HSA), using the equation described previously (Berezhkovskiy, 2007):

$$f_{u}\left(t\right)=\frac{1-\frac{\gamma P_{T}}{\frac{\left[{val}_{tot}\left(t\right)\right]}{BP}}-\frac{K_{d}}{\frac{\left[{val}_{tot}\left(t\right)\right]}{BP}}+\sqrt{{\left(1+\frac{\gamma P_{T}}{\frac{\left[{val}_{tot}\left(t\right)\right]}{BP}}+\frac{K_{d}}{\frac{\left[{val}_{tot}\left(t\right)\right]}{BP}}\right)}^{2}-4\frac{\gamma P_{T}}{\frac{\left[{val}_{tot}\left(t\right)\right]}{BP}}}}{2}$$ (1)

where $f_{u}\left(t\right)$ denotes concentration-dependent fraction of unbound VPA in plasma, $\gamma P_{T}$ denotes total albumin concentration (assumed constant) $,K_{d}$ denotes the drug binding affinity to HSA, ${val}_{tot}\left(t\right)$ denotes the total VPA concentration in blood, and BP denotes the blood to plasma ratio of VPA.

To verify the model against clinical data, we simulated a single intravenous dose of VPA infused over 1 h with 30 mg/kg or 130 mg/kg across 72 h in a population of healthy volunteers (Sim-Healthy, N = 8 for 10 trials) with 8% females to mirror the dosing and population of the clinical study (Georgoff et al., 2018). The Simcyp Simulator generates unique virtual individuals by sampling from a range of physiological parameters as described previously (Curry et al., 2024). The physiological parameters that vary between virtual individuals and are relevant to the mPBPK model for VPA are listed in Supplementary Table S3. In subsequent simulations, we predicted the systemic VPA concentration in a single population representative virtual individual of a ‘healthy’ individual following once-daily oral doses of 100, 200, 500, 1,000, 2,000, or 5,000 mg.

### 2.2 HEPATOKIN1 hepatocyte metabolism model reconstruction

We implemented a kinetic model of core hepatocyte metabolism, HEPATOKIN1 (Berndt et al., 2018), in Simcyp Designer software (V3; Certara Predictive Technologies, Sheffield, UK) (Matthews et al., 2023). Initially, the model implementation was verified to reproduce outcomes identical to those of the published model provisioned in SBML format by the authors (Supplementary Figure S1A), in line with previously recommended reproducibility assessments (Tiwari et al., 2021). Furthermore, the reconstructed model was evaluated for its ability to predict cytosolic glycogen concentration during the transition from the fasted (glucose = 4 mM) to the fed (glucose = 8 mM) state (Supplementary Figure S1B), as well as diurnal profiles of glutamate (Supplementary Figure S1C) and lactate (Supplementary Figure S1D) against experimental data, recovering the performance demonstrated by the published model (Berndt et al., 2018).

### 2.3 Integration of VPA mPBPK and HEPATOKIN1

Simcyp Designer software was used to integrate the mPBPK model for VPA, developed in the Simcyp Simulator, with the reconstructed large-scale model HEPATOKIN1 (hereafter referred to as the “integrated model”). The models were linked by predicted VPA systemic concentration, as illustrated in Figure 1. VPA is considered an exemplar Extended Clearance Classification System (ECCS) class 1A compound with high permeability, ∼90% contribution of metabolic hepatic clearance to systemic clearance, and no significant contribution of hepatic uptake or efflux transporters (Varma et al., 2015): hence, we assumed rapid equilibrium of unbound concentration between plasma and hepatic intracellular water. From the mPBPK predicted VPA systemic concentration, intracellular free VPA concentration ( ${val}_{cyt}$ ) within hepatocytes was estimated by considering factors such as plasma protein binding ( $\left.f_{u}\left(t\right)\right)$ , BP, and diffusion from plasma to the hepatocyte intracellular space (Supplementary Table S1 **)**. The equation to determine ${val}_{cyt}$ is described below:

$$\frac{\left.{d[val}_{cyt}\left(t\right)\right]}{dt}=D\left(\frac{{\left[val\right.}_{tot}\left(t\right)]f_{u}\left(t\right)}{BP}-{val}_{cyt}\left(t\right)\right)$$ (2)

where ${val}_{tot}$ denotes the total VPA concentration in blood, D denotes the diffusion rate between blood and hepatocytes of 3.6 h^-1^ (Berndt et al., 2018), and $f_{u}\left(t\right)$ is calculated using Equation 1.

The resulting *val*_*cyt*_ from Equation 2 then acts as a substrate to relevant metabolic enzymes within the metabolic model for metabolism and elimination. ${\;val}_{cyt}$ competitively inhibited the enzyme carnitine palmitoyltransferase I (CPT1) in accordance with previously established mechanisms (Berndt et al., 2018) and as reported previously in literature (Ezhilarasan and Mani, 2022; Aires et al., 2010) (Figure 1). In the model, CPT1 plays a vital role in the β-oxidation pathway as it is responsible for the elimination of endogenous fatty acids such as palmitate. The conversion of ${val}_{cyt}$ to valproyl coenzyme A $\left({valCoA}_{cyt}\right)$ by valproylcoa synthetase (VCS), followed by competitive inhibition of CPT1, is described in the equations below as represented in HEPATOKIN1. For all remaining equations and parameter definitions not explicitly shown here, please refer to the original HEPATOKIN1 model (Berndt et al., 2018).

$$\frac{\left.{d[valCoA}_{cyt}\left(t\right)\right]}{dt}=V_{\max}^{VCS}\frac{\left.{\left[val\right.}_{cyt}\left(t\right)\right]}{{\left[val\right.}_{cyt}\left(t\right)]+K_{m}^{{VCSval}_{cyt}}}\frac{\left.{\left[ATP\right.}_{cyt}\left(t\right)\right]}{{\left[ATP\right.}_{cyt}\left(t\right)]+K_{m}^{{VCSATP}_{cyt}}}\frac{\left.{\left[CoA\right.}_{cyt}\left(t\right)\right]}{{\left[CoA\right.}_{cyt}\left(t\right)]+K_{m}^{{VCSCoA}_{cyt}}}$$ (3)

Equation 3 describes conversion of val_cyt to valCoA_cyt where, V_max^VCS^ is the maximum velocity of VCS (3.6 mol/m^3.h^). $K_{m}^{{VCSval}_{cyt}}$ is the Michaelis constant for ${val}_{cyt}$ with VCS (1.65 mol/m^3^); $\left.{\left[ATP\right.}_{cyt}\left(t\right)\right]$ is the cytosolic ATP concentration, and the corresponding $K_{m}^{{VCSATP}_{cyt}}$ for ATP with VCS denotes its Michaelis constant (0.6 mol/m^3^); $\left.{\left[CoA\right.}_{cyt}\left(t\right)\right]$ is the cytosolic coenzyme A concentration, and the corresponding $K_{m}^{{VCSCoA}_{cyt}}$ for coenzyme A with VCS (0.0025 mol/m^3^) denotes it Michaelis constant.

$$v_{CPT1}=V_{\max}^{CPT1}\frac{\left[{c16CoA}_{cyt}\left(t\right)\right]}{\left[{c16CoA}_{cyt}\left(t\right)\right]+K_{m}^{{c16CoA}_{cyt}}}\frac{\left[{carnitine}_{cyt}\left(t\right)\right]}{\left[{carnitine}_{cyt}\left(t\right)\right]+K_{m}^{{carnitine}_{cyt}}}$$ (4)

Equation 4 describes $v_{CPT1}$ , which is the flux of CPT1 (mol/m^3^.h) enzyme driven removal rate of cytosolic palmitate coenzyme A $\left.{\left[c16CoA\right.}_{cyt}\left(t\right)\right]$ . $V_{\max}^{CPT1}$ is the maximum velocity of CPT1 (1.8 mol/m^3^.h). Cytosolic carnitine, $\left.{\left[carnitine\right.}_{cyt}\left(t\right)\right]$ , is the CPT1 substrate concentration, and $K_{m}^{{carnitine}_{cyt}}$ is the Michaelis constant for carnitine with CPT1 (0.032 mol/m^3^). $K_{m}^{{c16CoA}_{cyt}}$ is the Michaelis constant for $\left.{\left[c16CoA\right.}_{cyt}\left(t\right)\right]$ with CPT1, calculated using Equation 5 below to describe VPA-mediated inhibition.

$$K_{m}^{{c16CoA}_{cyt}}=K_{0}^{{c16CoA}_{cyt}}\left(1+\frac{\left.{\left[valCoA\right.}_{cyt}\left(t\right)\right]}{\;K_{i}^{{valCoA}_{cyt}}}\right)\left(1+\frac{\left[{malCoA2}_{imm}\left(t\right)\right]}{K_{i}^{{malCoA2}_{imm}}}\right)$$ (5)

$K_{0}^{{c16CoA}_{cyt}}$ is the basal Michaelis constant for ${\left[c16CoA\right.}_{cyt}\left(t\right)]$ binding with CPT1 (0.03 mol/m^3^), which is scaled by competitive inhibition from $\left.{\left[valCoA\right.}_{cyt}\left(t\right)\right]$ and inhibitory constant $K_{i}^{{valCoA}_{cyt}}$ (0.057 mol/m^3^) and malonyl coenzyme A (malCoA2) $\left[{malCoA2}_{imm}\left(t\right)\right]$ and inhibitory constant $K_{i}^{{malCoA2}_{imm}}$ (0.0025 mol/m^3^).

The integrated model was simulated with repeated oral doses of VPA, ranging from 100 to 5,000 mg, at specific dose levels of 100, 200, 500, 1,000, 2,000, and 5,000 mg. Doses of 100 mg, 1,000 mg, and 5,000 mg were selected as representative scenarios for low, medium, and high dosing levels. This dose selection was based on an initial recommended dose range of 10–15 mg/kg/day (FDA, 2016) and the absence of safety guidelines for valproate doses exceeding 60 mg/kg/day (FDA, 2016). The integrated model was simulated over a 5-day (120 h) period, beginning with an initial 24-h phase without VPA dosing to allow core metabolites in HEPATOKIN1 to stabilize in a steady state. This was followed by a regimen of once-daily oral VPA administration for the remaining 4 days (96 h).

### 2.4 PPARα-mediated regulation

We identified metabolic reactions within the metabolic component of the integrated model that are reported in the literature to be regulated by the transcription factor PPARα (Supplementary Table S2). The flux of these metabolic reactions was scaled by estimating the levels of ‘activated’ PPARα using Equation 6 below. Here, we assume that PPARα activation is solely driven by cytosolic palmitate ( $\left.{c16}_{cyt}\right)$ . Although oleic acid is recognised as the most abundant fatty acid in healthy human liver, palmitic acid is most prominently associated with adverse metabolic outcomes, including dyslipidaemia, and hyperglycaemia (Kotronen et al., 2010; Carta et al., 2017). This simplified assumption, consistent with HEPATOKIN1, was adopted to manage the complexity of modelling fatty acid species and to exemplify VPA-induced hepatotoxicity. It was modelled using a Hill function, as described: the maximum production rate, $\Phi_{\max}=0.5\;h^{-1}$ (assuming average human protein production rate (Shamir et al., 2016)); the half-maximal ${c16}_{cyt}$ concentration, $\Phi_{50}=5\times 10^{-5}\;mM$ (arbitrarily chosen), Hill’s coefficient, n=0.2 (arbitrarily chosen), and the PPARα degradation rate $\delta_{PPAR\alpha }=0.1008\;h^{-1}$ (assuming average human protein degradation rate (Eden et al., 2011)).

$$\frac{dPPAR\alpha }{dt}=\frac{\Phi_{\max}{c16}_{cyt}^{n}}{\Phi_{50}^{n}+{c16}_{cyt}^{n}}-\delta_{PPAR\alpha }PPAR\alpha$$ (6)

The values for $\Phi_{50}$ and Hill’s coefficient were arbitrarily chosen due to the absence of experimental data to inform a data-driven value. Hence, we performed a sensitivity analysis on both parameters within reasonable ranges informed by diurnal concentrations from HEPTAOKIN1 to assess the impact of varying these parameters on the PPARα profile at baseline values in the absence of VPA dosing (Supplementary Figures S2, S3). Varying $\Phi_{50}$ between $1\times 10^{-4}$ and $1\times 10^{-6}\;mM$ concentrations (basal ${c16}_{cyt}$ concentrations fluctuate between $1.57\times 10^{-5}$ and $5.56\times 10^{-6}\;mM$ ) invariably led to an increase in PPARα values above 1, regardless of the specific value selected. However, when we examined Hill’s coefficient values between 0 and 0.8, not all values for Hill’s coefficient resulted in PPARα values exceeding 1; for example, at n=0.8 , parts of the PPARα profile fell below 1, implying a reversal in the direction of the PPARα ’s scaling effect on enzymes. Since our assumption is that PPARα exerts a linear scaling effect on enzyme kinetics, we chose a Hill’s coefficient of 0.2 that consistently maintained PPARα values above one to ensure the directionality of upregulation and downregulation on affected enzymes. Although variations in these values may result in quantitatively distinct PPARα levels, the directional change in target regulation and the ensuing qualitative effects are expected to remain unchanged.

## 3 Results

### 3.1 Impact of VPA dosing on lipid metabolism without PPARα-mediated regulation

To ensure human relevance of the integrated model, we devised a human PBPK framework to model VPA pharmacokinetics, which was verified against observed human data. This human relevance stems partly from the incorporation of physiology-based parameters sourced from literature, including the blood to plasma ratio and human serum albumin binding affinity (see Methods). Consistent with its human relevance, the VPA PBPK model quantitatively replicates clinically observed plasma concentrations of VPA following intravenous doses of 30 and 130 mg/kg administered over 1 h (Supplementary Figure S4). For instance, the model’s predicted mean and 95% confidence interval, which incorporates population variability in physiology, recovers the measured observations well, such as peak concentration ( $C_{\max}$ ) and subsequent elimination from plasma. Following this validation, the model’s predicted systemic VPA concentration was used to calculate the free VPA concentration in hepatocytes. This effective concentration was then applied to stimulate the HEPATOKIN1 model within the integrated model, thereby enhancing its relevance to human liver tissue.

**FIGURE 2:**
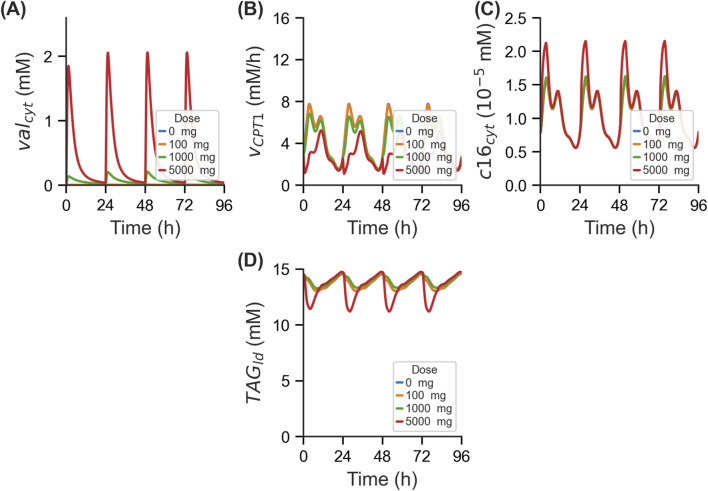
VPA induced changes in lipid metabolism. Time-course profiles in single population representative virtual individual for **(A)** free hepatocellular concentration of VPA (val_cyt_), **(B)** flux of CPT1 enzyme reaction (v_CPT1_), **(C)** concentration of cytosolic palmitate (c16_cyt_), and **(D)** concentration of triglyceride content (TAG_ld_). CPT1, carnitine palmitoyltransferase 1.

This integrated model was employed to investigate the effects of increasing doses of VPA on lipid metabolism. We simulated the model with oral VPA administration once every day over a 72-h period in a single population representative virtual individual, as detailed in the Methods section. As expected, intracellular concentration of VPA within hepatocytes ( ${val}_{cyt}$ ) levels rose with higher VPA doses (Figure 2A). Notably, carnitine palmitoyltransferase 1 (CPT1) flux, $v_{CPT1}$ , decreased with increased VPA dosing due to the competitive inhibition by VPA (Figure 2B), resulting in elevated concentrations of cytosolic palmitate, $c16_{cyt}$ (Figure 2C). Since palmitate is a cofactor of TAG, including those found in lipid droplets ( $TAG_{ld}$ ) (Figure 2D), increasing VPA dosing generally led to elevated $TAG_{ld}$ levels. Surprisingly, at the highest evaluated dose of 5,000 mg, a reduction in $TAG_{ld}$ was predicted, suggesting a possible threshold effect or alternative metabolic regulation at extremely high VPA concentrations occurring from the integrated model.

To elucidate the mechanism responsible for the reduction in $TAG_{ld}$ predicted at higher doses, we investigated additional TAG cofactors such as cytosolic long chain acyl coenzyme A ( $c16CoA_{cyt}$ ) (Supplementary Figure S5A) and cytosolic coenzyme A ( $CoA_{cyt}$ ) (Supplementary Figure S5B). In simulations at the highest dose of 5,000 mg, $CoA_{cyt}$ reduced to near zero concentrations, indicating complete depletion of the cofactor. This is likely contributing to the reduction of $TAG_{ld}$ below baseline levels predicted only at high doses as the cofactor is completely depleted to make TAG. However, it is physiologically improbable for metabolites to be consistently depleted, as feedback mechanisms would typically prevent this; indeed, elevated TAG levels are reported to increase monotonically with VPA dosing (Bai et al., 2017; Xu et al., 2019). Therefore, the paradoxical reduction in $TAG_{ld}$ at high VPA dosing is likely due to the HEPATOKIN1 model not accounting for these physiological feedback mechanisms, particularly gene-regulatory responses, that are presumably activated under high-dose conditions.

### 3.2 Incorporation of PPARα-mediated regulation is essential to predict monotonic increase in lipids

**FIGURE 3:**
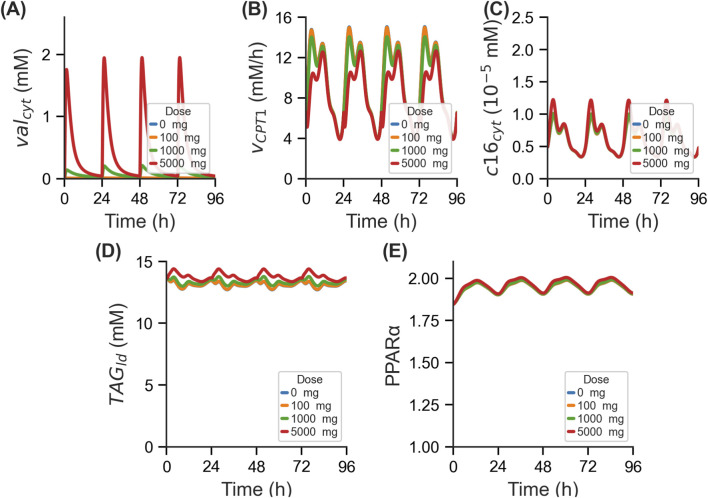
Effect of PPARα-mediated regulation on VPA induced changes in lipid metabolism. Time-course profiles in a single population representative virtual individual for **(A)** free hepatocellular concentration of VPA (val_cyt_), **(B)** flux of CPT1 enzyme reaction (v_CPT1_), **(C)** concentration of cytosolic palmitate (c16_cyt_), **(D)** concentration of triglyceride content (TAG_ld_), and **(E)** transcription factor PPARα regulation with various repeated intravenous dosing of VPA. PPARα, peroxisome proliferator-activated receptor α; VPA, valproic acid.

Next, to demonstrate the necessity of incorporating feedback mechanisms into our integrated model, we examined the role of the transcription factor PPARα, given its critical functions in regulating fatty acid and lipid metabolism (Maldonado et al., 2018). We semi-quantitatively modelled the impact of PPARα exclusively on the metabolic enzymes it regulates and subsequently performed simulations in a single population representative virtual individual with varying VPA doses (see Methods). As expected, integrating PPARα mechanism revealed no alterations in the ${val}_{cyt}$ dynamics compared to the outcomes without it, as shown in Figure 3A. We also observed a dose-dependent decrease in $v_{CPT1}$ as expected, attributed to competitive inhibition from rising ${val}_{cyt}$ levels (Figure 3B). However, the inclusion of PPARα resulted in higher quantitative levels of $v_{CPT1}$ across all doses than those predicted without PPARα (Figure 2B) likely from CPT1 upregulation by PPARα . Similarly, there was a dose-dependent increase in $c16_{cyt\;}$ (Figure 3C) due to reduced $v_{CPT1}$ , yet the levels were quantitatively lower than those simulated without PPARα (Figure 2C). Surprisingly, the incorporation of PPARα ensured a consistent dose-dependent increase in $TAG_{ld}$ with escalating VPA doses (Figure 3D), a behaviour absent when PPARα was not included. It is important to mention that the predicted hepatic $TAG_{ld}$ concentrations are roughly 10-fold higher than the TAG concentrations observed in patients’ plasma (typically ∼1.3–1.8 mM) (Sidhu et al., 2017; Aksoy D et al., 2015). The reason may stem from the larger pool of TAG in the liver compared to that present in plasma (Farquhar et al., 1965). While the model includes packaging of TAG into very low density lipoprotein (VLDL) and hepatic VLDL secretion, it does not currently predict plasma TAG. Incorporating systemic distribution, peripheral uptake, and plasma clearance kinetics, along with refining related parameterization, would enable prediction of plasma TAG and may help reconcile the higher hepatic $TAG_{ld}$ simulations with observed plasma measurements.

The predicted baseline for PPARα remains in the range of 1.9–2.0, even in the absence of VPA (Figure 3E), likely due to diurnal variations of $c16_{cyt}$ under physiological conditions, highlighting an active basal level of gene regulation essential for maintaining homeostasis. Although the increase in PPARα with rising VPA doses is modest, it is sufficient to elicit a consistent dose-dependent response in metabolite levels. Moreover, examining the cofactors of $TAG_{ld}$ with the inclusion of PPARα reveals distinct dose-dependent dynamics: $c16CoA_{cyt}$ increasing (Supplementary Figure S5C), while $CoA_{cyt\;}$ decreasing correspondingly (Supplementary Figure S5D). Notably, the integration of PPARα successfully addresses the complete depletion of the cofactor $CoA_{cyt\;}$ , a limitation previously observed in model simulations lacking PPARα regulation. This adjustment underscores the crucial role of PPARα in maintaining cofactor stability across varying VPA dosages.

**FIGURE 4:**
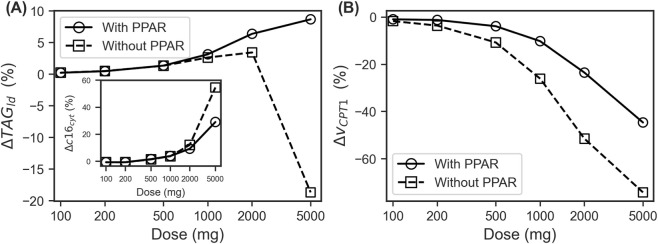
Dose-response curves of maximum % changes in **(A)** lipid droplet TAG content (Δ TAG_ld_) with a figure inset of the dose-response curve of % changes in maximum cytosolic palmitate concentrations (Δ c16_cyt_) and **(B)** maximum % changes in CPT1 enzyme velocity (Δ v_CPT1_) compared to no dose outcomes with increasing doses of once daily valproic acid. CPT1, carnitine palmitoyltransferase 1.

Expanding upon these findings, we evaluated the maximum relative change in $TAG_{ld}$ for various VPA dosages both with and without PPARα , compared to a no-treatment scenario. This analysis involved determining the relative change at each time point between treated and untreated conditions for each dose and plotting the maximum predicted relative change for each dosage. The dose-dependent responses, summarized in Figure 4A, indicate that model predictions incorporating PPARα retain a consistent increase in $TAG_{ld}$ across dosages, while predictions without PPARα exhibited a marked reduction in the percentage change in maximum $TAG_{ld}$ content at 5,000 mg VPA. This model behaviour holds even as both models show a consistent dose-dependent increase in the percentage change in maximum $c16_{cyt}$ (Figure 4A inset). Furthermore, as shown in Figure 4B, both models predict a dose-dependent reduction in $v_{CPT1}$ , owing to the competitive inhibition of VPA. However, the reduction in $v_{CPT1}$ is noticeably less pronounced in the model with PPARα at doses 1,000 mg and above compared to those without. This difference can be explained by the upregulation of CPT1 by PPARα , suggesting PPARα mitigates the reduction in $v_{CPT1}$ resulting from VPA inhibition.

## 4 Discussion

This study aimed to apply and extend the hepatocyte metabolism model for assessing early markers of liver steatosis, focusing on CPT1 inhibition by VPA and evaluating dose-dependent changes in various hepatic metabolites. We integrated three different modelling components: PBPK, a large-scale hepatocyte metabolism model (HEPATOKIN1), and PPARα-mediated regulation, forming a tool-based and multiscale QST model. PBPK integration enabled the simulation of physiologically relevant VPA concentrations, driving downstream effects within the hepatocyte metabolism model. Our findings demonstrated that competitive CPT1 inhibition of VPA alone could induce dose-dependent increases in lipids, including TAG, as clinically observed before (Nanau and Neuman, 2013). However, the addition of PPARα regulation into the hepatocyte metabolism model was essential to address the paradoxical reduction of liver metabolites at high VPA doses, underscoring the importance of considering known biological regulations likely to be activated under extreme scenarios such as high drug dosing.

An increase in circulating TAG has been observed in epileptic patients undergoing VPA treatment, with reported increases ranging from approximately 10%–50% of circulating TAG (Sidhu et al., 2017; Aksoy D et al., 2015). In contrast, our model predicts TAG within hepatocyte lipid droplets, with a maximum TAG increase of about 10% (Figure 4A). This discrepancy may arise from several factors, including comparing circulating TAG against TAG within hepatocyte lipid droplets, patient demographics (such as age (Guo et al., 2022) and BMI), treatment duration, and the presence of insulin resistance (Ozdemir et al., 2011; Pylvanen et al., 2003). The reported increases in TAG due to VPA treatment typically manifest after 1–2 years, whereas our simulations only spanned a few days. Prolonged treatment durations may have unforeseen implications on additional metabolic pathways or regulatory networks. Furthermore, many patients receiving VPA monotherapy develop insulin resistance, which can significantly impact metabolic pathways (Sidhu et al., 2017; Pylvanen et al., 2003). Our model does not account for the influence of insulin on TAG synthesis, which may exacerbate effects within the metabolic network and explain the higher observed changes than predicted in our simulations.

There are additional limitations associated with the current implementation of the integrated model that must be recognized. The exploration of PPARα-driven regulation in this study is not comprehensive, due to the complex and wide-spread influence of PPARα (Kersten, 2014), as well as uncertainties surrounding its effects on specific enzyme kinetics. Additionally, there is a paucity of quantitative data on PPARα effects, and human-specific data is even more limited (Kersten and Stienstra, 2017), which creates a challenge in comprehensively modelling the regulatory effects of PPARα on individual enzymes. An important interspecies limitation is that PPARα activation in rodents is generally higher than in humans, with more pronounced transcriptional responses and metabolic modelling reported in rodents (McMullen et al., 2020). Consequently, extrapolating PPARα-mediated any insights from rodent studies to clinical observations warrants caution in interpretation. Nevertheless, our model provides a framework that can be re-parameterized with human-specific data to support cross-species translation, and the integration of a human VPA PBPK model validated against clinical data enables exploration of human-driven PK effects as a first step toward such translation. Similar challenges apply to the hepatocyte metabolism model, which relies on chimeric data comprising inputs from various preclinical studies and species to inform or optimise parameters. Consequently, our focus was on the qualitative direction of effects when interpreting the impact of VPA, rather than precise quantitative predictions.

In this study, we focus on PPARα-driven regulatory effects, given the extensive research linking PPARα specifically to liver pathways (Jump et al., 2013). Consideration of other regulatory pathways fell outside the scope of this study. However, it is important to acknowledge that numerous other regulatory pathways can influence metabolic pathways in the hepatocyte metabolism model. These include other PPAR subtypes (PPARβ/δ, PPARγ) (Kersten and Stienstra, 2017), other transcription factors (Jump et al., 2013; Han et al., 2016; He et al., 2025) such as HNF4, retinoid X-receptor, liver X receptor, NRF2, and proteins (Yamashita et al., 2001) like ChRE-binding protein. Therefore, when utilizing this QST model or similar models, it is essential to evaluate the relevance of additional regulatory pathways pertinent to the specific research hypothesis and adapt the model accordingly.

The limitations discussed above can be addressed through further quantitative measurements in specific metabolic pathways, which would enhance the translatability and clinical relevance of the QST model. For instance, advancing our understanding of liver metabolism pathways specific to humans, with quantitative outcomes, can provide direct insights for informing parameters or optimizing them to replace those derived from preclinical species. This refinement would significantly improve the model’s applicability in clinical settings. Moreover, incorporating additional regulatory pathways involves identifying the relevant liver metabolism pathways affected, determining the direction of regulation (up or down), and establishing connections to relevant metabolites, similar to the inclusion of PPARα included in this work. To further increase confidence and applicability, integration of clinically relevant, non-invasive DILI biomarkers will be important to enable prospective validation of model predictions. In parallel, expanding the set of test compounds with available clinical data will help reduce parameter uncertainty, improve identifiability, and strengthen the robustness of quantitative estimates. Therefore, as our understanding of VPA effects and hepatocyte metabolism, along with regulatory effects, continues to expand, the model demonstrated here should be iteratively updated to incorporate relevant mechanisms essential for exploring specific research hypotheses.

This work demonstrates the potential of multi-scale *in silico* modelling as part of the new approach methodologies (NAMs) landscape. By integrating PBPK, a large-scale hepatocyte metabolism model, and PPARα-mediated regulation into a QST model, this study demonstrates a mechanistic, human-relevant approach to predicting adverse outcomes. This computational framework offers a powerful alternative to traditional animal testing, focusing on understanding the underlying biological mechanisms of toxicity (e.g., CPT1 inhibition) through the integration of multi-modal data and striving for greater translatability to human health. Such integrated modelling efforts are crucial for enhancing early hazard identification, refining risk assessment, and ultimately reducing reliance on animal models in toxicology and drug development.

## Data Availability

Simcyp Designer and Simcyp Simulator model workspaces to generate VPA effects on hepatic metabolism, Simcyp Simulator workspaces for VPA validation, and their curated Excel outputs are available on the platform BioModels (Malik-Sheriff et al., 2020) as MODEL2509090001. Simcyp Designer and Simcyp Simulator software can be licensed from Certara Predictive Technologies, and a license to reproduce outcomes can be provided upon request.

## References

[B1] Ahangar N. Naderi M. Noroozi A. Ghasemi M. Zamani E. Shaki F. (2017). Zinc deficiency and oxidative stress involved in valproic acid induced hepatotoxicity: protection by zinc and selenium supplementation. Biol. Trace Elem. Res. 179, 102–109. 10.1007/s12011-017-0944-z 28124216

[B2] Aires C. C. Ijlst L. Stet F. Prip-Buus C. DE Almeida I. T. Duran M. (2010). Inhibition of hepatic carnitine palmitoyl-transferase I (CPT IA) by valproyl-CoA as a possible mechanism of valproate-induced steatosis. Biochem. Pharmacol. 79, 792–799. 10.1016/j.bcp.2009.10.011 19854160

[B3] Aksoy D S. V. Çevik B. Pekdaş E. Kurt S. (2015). Serum lipids and thyroid functions in young epileptic patients undergoing monotherapy with valproate or levetiracetam. Eur. J. Gen. Med. 12, 59–63. 10.15197/sabad.1.12.10

[B4] Ashworth W. B. Davies N. A. Bogle I. D. (2016). A computational model of hepatic energy metabolism: understanding zonated damage and steatosis in NAFLD. PLoS Comput. Biol. 12, e1005105. 10.1371/journal.pcbi.1005105 27632189 PMC5025084

[B5] Bai X. Hong W. Cai P. Chen Y. Xu C. Cao D. (2017). Valproate induced hepatic steatosis by enhanced fatty acid uptake and triglyceride synthesis. Toxicol. Appl. Pharmacol. 324, 12–25. 10.1016/j.taap.2017.03.022 28366540

[B6] Barbier O. Torra I. P. Sirvent A. Claudel T. Blanquart C. Duran-Sandoval D. (2003). FXR induces the UGT2B4 enzyme in hepatocytes: a potential mechanism of negative feedback control of FXR activity. Gastroenterology 124, 1926–1940. 10.1016/s0016-5085(03)00388-3 12806625

[B7] Battista C. Howell B. A. Siler S. Q. Watkins P. B. (2018). “An introduction to DILIsym® software, a mechanistic mathematical representation of drug-induced liver injury,” in Drug-induced liver toxicity. Editors CHEN M. WILL Y. (New York, NY: Springer New York).

[B8] Beattie K. A. Verma M. Brennan R. J. Clausznitzer D. Damian V. Leishman D. (2024). Quantitative systems toxicology modeling in pharmaceutical research and development: an industry‐wide survey and selected case study examples. CPT Pharmacometrics and Syst. Pharmacol. 13, 2036–2051. 10.1002/psp4.13227 39412216 PMC11646944

[B9] Berezhkovskiy L. M. (2007). On the calculation of the concentration dependence of drug binding to plasma proteins with multiple binding sites of different affinities: determination of the possible variation of the unbound drug fraction and calculation of the number of binding sites of the protein. J. Pharm. Sci. 96, 249–257. 10.1002/jps.20777 17051587

[B10] Berndt N. Bulik S. Wallach I. Wünsch T. König M. Stockmann M. (2018). HEPATOKIN1 is a biochemistry-based model of liver metabolism for applications in medicine and pharmacology. Nat. Communications 9, 2386. 10.1038/s41467-018-04720-9 29921957 PMC6008457

[B11] Carta G. Murru E. Banni S. Manca C. (2017). Palmitic acid: physiological role, metabolism and nutritional implications. Front. Physiol. 8, 902. 10.3389/fphys.2017.00902 29167646 PMC5682332

[B12] Chang T. K. Abbott F. S. (2006). Oxidative stress as a mechanism of valproic acid-associated hepatotoxicity. Drug Metab. Rev. 38, 627–639. 10.1080/03602530600959433 17145692

[B13] Curry L. Alrubia S. Bois F. Y. Clayton R. EL-Khateeb E. Johnson T. N. (2024). A guide to developing population files for physiologically-based pharmacokinetic modeling in the simcyp simulator. CPT Pharmacometrics Syst. Pharmacol. 13, 1429–1447. 10.1002/psp4.13202 39030888 PMC11533108

[B14] Dreyer C. Keller H. Mahfoudi A. Laudet V. Krey G. Wahli W. (1993). Positive regulation of the peroxisomal beta-oxidation pathway by fatty acids through activation of peroxisome proliferator-activated receptors (PPAR). Biol. Cell 77, 67–76. 10.1016/s0248-4900(05)80176-5 8390886

[B15] Dutta-Moscato J. Solovyev A. Mi Q. Nishikawa T. Soto-Gutierrez A. Fox I. J. (2014). A multiscale agent-based *in silico* model of liver fibrosis progression. Front. Bioeng. Biotechnol. 2, 18. 10.3389/fbioe.2014.00018 25152891 PMC4126446

[B16] Eden E. Geva-Zatorsky N. Issaeva I. Cohen A. Dekel E. Danon T. (2011). Proteome half-life dynamics in living human cells. Sci. (New York, N.Y.) 331, 764–768. 10.1126/science.1199784 21233346

[B17] Eichenbaum G. Yang K. Gebremichael Y. Howell B. A. Murray F. J. Jacobson-Kram D. (2020). Application of the DILIsym(R) quantitative systems toxicology drug-induced liver injury model to evaluate the carcinogenic hazard potential of acetaminophen. Regul. Toxicol. Pharmacol. 118, 104788. 10.1016/j.yrtph.2020.104788 33153971

[B18] EL-Khateeb E. Burkhill S. Murby S. Amirat H. Rostami-Hodjegan A. Ahmad A. (2021). Physiological-based pharmacokinetic modeling trends in pharmaceutical drug development over the last 20-years; in-depth analysis of applications, organizations, and platforms. Biopharm. Drug Dispos. 42, 107–117. 10.1002/bdd.2257 33325034

[B19] Escher S. Limonciel A. Jennings P. VAN Vugt-Lussenburg A. Burg E. Mombelli E. (2020). Case study on the use of integrated approaches to testing and assessment for prediction of a 90 day repeated dose toxicity study (OECD 408) for 2-ETHYLBUTYRIC acid using a read-across approach from other branched carboxylic acids. OECD.

[B20] Ezhilarasan D. Mani U. (2022). Valproic acid induced liver injury: an insight into molecular toxicological mechanism. Environ. Toxicol. Pharmacol. 95, 103967. 10.1016/j.etap.2022.103967 36058508

[B21] Ezuruike U. Zhang M. Pansari A. DE Sousa Mendes M. Pan X. Neuhoff S. (2022). Guide to development of compound files for PBPK modeling in the simcyp population-based simulator. CPT Pharmacometrics Syst. Pharmacol. 11, 805–821. 10.1002/psp4.12791 35344639 PMC9286711

[B22] Farquhar J. W. Gross R. C. Wagner R. M. Reaven G. M. (1965). Validation of an incompletely coupled two-compartment nonrecycling catenary model for turnover of liver and plasma triglyceride in man. J. Lipid Res. 6, 119–134. 10.1016/S0022-2275(20)39649-8 14280459

[B23] FDA (2016). DEPAKENE (valproic acid) label. Available online at: https://www.accessdata.fda.gov/drugsatfda_docs/label/2016/018081s065_018082s048lbl.pdf (Accessed February 02, 2023).

[B24] Georgoff P. E. Nikolian V. C. Bonham T. Pai M. P. Tafatia C. Halaweish I. (2018). Safety and tolerability of intravenous valproic acid in healthy subjects: a phase I dose-escalation trial. Clin. Pharmacokinet. 57, 209–219. 10.1007/s40262-017-0553-1 28497259 PMC5699961

[B25] Gille C. Bolling C. Hoppe A. Bulik S. Hoffmann S. Hubner K. (2010). HepatoNet1: a comprehensive metabolic reconstruction of the human hepatocyte for the analysis of liver physiology. Mol. Syst. Biol. 6, 411. 10.1038/msb.2010.62 20823849 PMC2964118

[B26] Goldring C. E. Russomanno G. Pin C. Trairatphisan P. Beattie K. A. Fisher C. P. (2025). Quantitative systems toxicology: modelling to mechanistically understand and predict drug safety. Nat. Rev. Drug Discov. 25, 138–153. 10.1038/s41573-025-01308-z 41145622

[B27] Guo H. L. Dong N. Chen F. Zeng Y. Y. Hu Y. H. Xia Y. (2022). Effect of long-term valproic acid therapy on lipid profiles in paediatric patients with epilepsy: a meta-analysis. Epileptic Disord. 24, 822–830. 10.1684/epd.2022.1460 35816100

[B28] Gutgesell A. Wen G. Konig B. Koch A. Spielmann J. Stangl G. I. (2009). Mouse carnitine-acylcarnitine translocase (CACT) is transcriptionally regulated by PPARalpha and PPARdelta in liver cells. Biochim. Biophys. Acta 1790, 1206–1216. 10.1016/j.bbagen.2009.06.012 19577614

[B29] Han H. S. Kang G. Kim J. S. Choi B. H. Koo S. H. (2016). Regulation of glucose metabolism from a liver-centric perspective. Exp. Mol. Med. 48, e218. 10.1038/emm.2015.122 26964834 PMC4892876

[B30] He X. Yuan R. Chen Y. Huang W. Xu Z. Wang B. (2025). Mechanism of valproic acid-induced hepatic steatosis *via* enhancing NRF2-FATP2-mediated fatty acid uptake. Theranostics 15, 5258–5276. 10.7150/thno.108593 40303331 PMC12036889

[B31] Jones H. Rowland-Yeo K. (2013). Basic concepts in physiologically based pharmacokinetic modeling in drug discovery and development. CPT Pharmacometrics Syst. Pharmacol. 2, e63. 10.1038/psp.2013.41 23945604 PMC3828005

[B32] Jump D. B. Tripathy S. Depner C. M. (2013). Fatty acid-regulated transcription factors in the liver. Annu. Rev. Nutr. 33, 249–269. 10.1146/annurev-nutr-071812-161139 23528177 PMC3940310

[B33] Kanwal F. Neuschwander-Tetri B. A. Loomba R. Rinella M. E. (2024). Metabolic dysfunction-associated steatotic liver disease: update and impact of new nomenclature on the American association for the study of liver diseases practice guidance on nonalcoholic fatty liver disease. Hepatology 79, 1212–1219. 10.1097/HEP.0000000000000670 38445559 PMC13435502

[B34] Ke A. Barter Z. Rowland-Yeo K. Almond L. (2016). Towards a best practice approach in PBPK modeling: case example of developing a unified efavirenz model accounting for induction of CYPs 3A4 and 2B6. CPT Pharmacometrics Syst. Pharmacol. 5, 367–376. 10.1002/psp4.12088 27435752 PMC4961080

[B35] Kersten S. (2014). Integrated physiology and systems biology of PPARalpha. Mol. Metab. 3, 354–371. 10.1016/j.molmet.2014.02.002 24944896 PMC4060217

[B36] Kersten S. Stienstra R. (2017). The role and regulation of the peroxisome proliferator activated receptor alpha in human liver. Biochimie 136, 75–84. 10.1016/j.biochi.2016.12.019 28077274

[B37] Kotronen A. Seppanen-Laakso T. Westerbacka J. Kiviluoto T. Arola J. Ruskeepaa A. L. (2010). Comparison of lipid and fatty acid composition of the liver, subcutaneous and intra-abdominal adipose tissue, and serum. Obes. (Silver Spring) 18, 937–944. 10.1038/oby.2009.326 19798063

[B38] Lagace D. C. O'Brien W. T. Gurvich N. Nachtigal M. W. Klein P. S. (2004). Valproic acid: how it works. Or not. Clin. Neurosci. Res. 4, 215–225. 10.1016/j.cnr.2004.09.013

[B39] Lakhani V. V. Generaux G. Howell B. A. Longo D. M. Watkins P. B. (2023). Assessing liver effects of cannabidiol and valproate alone and in combination using quantitative systems toxicology. Clin. Pharmacol. Ther. 114, 1006–1014. 10.1002/cpt.3004 37458709

[B40] Laudadio I. Manfroid I. Achouri Y. Schmidt D. Wilson M. D. Cordi S. (2012). A feedback loop between the liver-enriched transcription factor network and miR-122 controls hepatocyte differentiation. Gastroenterology 142, 119–129. 10.1053/j.gastro.2011.09.001 21920465

[B41] Li H. H. Tyburski J. B. Wang Y. W. Strawn S. Moon B. H. Kallakury B. V. (2014). Modulation of fatty acid and bile acid metabolism by peroxisome proliferator-activated receptor alpha protects against alcoholic liver disease. Alcohol Clin. Exp. Res. 38, 1520–1531. 10.1111/acer.12424 24773203 PMC4047177

[B42] Maldonado E. M. Fisher C. P. Mazzatti D. J. Barber A. L. Tindall M. J. Plant N. J. (2018). Multi-scale, whole-system models of liver metabolic adaptation to fat and sugar in non-alcoholic fatty liver disease. NPJ Syst. Biol. Appl. 4, 33. 10.1038/s41540-018-0070-3 30131870 PMC6102210

[B43] Malik-Sheriff R. S. Glont M. Nguyen T. V. N. Tiwari K. Roberts M. G. Xavier A. (2020). BioModels-15 years of sharing computational models in life science. Nucleic Acids Res. 48, D407–D415. 10.1093/nar/gkz1055 31701150 PMC7145643

[B44] Matthews R. J. Hollinshead D. Morrison D. VAN DER Graaf P. H. Kierzek A. M. (2023). QSP designer: quantitative systems pharmacology modeling with modular biological process map notation and multiple language code generation. CPT Pharmacometrics and Syst. Pharmacol. 12, 889–903. 10.1002/psp4.12972 37452454 PMC10349184

[B45] Mcmullen P. D. Bhattacharya S. Woods C. G. Pendse S. N. Mcbride M. T. Soldatow V. Y. (2020). Identifying qualitative differences in PPARalpha signaling networks in human and rat hepatocytes and their significance for next generation chemical risk assessment methods. Toxicol Vitro 64, 104463. 10.1016/j.tiv.2019.02.017 31628012

[B46] Minnich A. Tian N. Byan L. Bilder G. (2001). A potent PPARalpha agonist stimulates mitochondrial fatty acid beta-oxidation in liver and skeletal muscle. Am. J. Physiol. Endocrinol. Metab. 280, E270–E279. 10.1152/ajpendo.2001.280.2.E270 11158930

[B47] Nanau R. M. Neuman M. G. (2013). Adverse drug reactions induced by valproic acid. Clin. Biochem. 46, 1323–1338. 10.1016/j.clinbiochem.2013.06.012 23792104

[B48] Ozdemir O. Yakut A. Dinleyici E. C. Aydogdu S. D. Yarar C. Colak O. (2011). Serum asymmetric dimethylarginine (ADMA), homocysteine, vitamin B(12), folate levels, and lipid profiles in epileptic children treated with valproic acid. Eur. J. Pediatr. 170, 873–877. 10.1007/s00431-010-1366-5 21140275

[B49] Pylvanen V. Knip M. Pakarinen A. J. Turkka J. Kotila M. Rattya J. (2003). Fasting serum insulin and lipid levels in men with epilepsy. Neurology 60, 571–574. 10.1212/01.wnl.0000048209.07526.86 12601094

[B50] Qian H. Deng X. Huang Z. W. Wei J. Ding C. H. Feng R. X. (2015). An HNF1alpha-regulated feedback circuit modulates hepatic fibrogenesis *via* the crosstalk between hepatocytes and hepatic stellate cells. Cell Res. 25, 930–945. 10.1038/cr.2015.84 26169608 PMC4528057

[B51] Rowland Yeo K. Gil Berglund E. Chen Y. (2024). Dose optimization informed by PBPK modeling: state-of-the art and future. Clin. Pharmacol. Ther. 116, 563–576. 10.1002/cpt.3289 38686708

[B52] Schleicher J. Dahmen U. (2018). Computational modeling of oxidative stress in fatty livers elucidates the underlying mechanism of the increased susceptibility to ischemia/reperfusion injury. Comput. Struct. Biotechnol. J. 16, 511–522. 10.1016/j.csbj.2018.10.013 30505404 PMC6247397

[B53] Shamir M. Bar-On Y. Phillips R. Milo R. (2016). SnapShot: timescales in cell biology. Cell 164, 1302–1302.e1. 10.1016/j.cell.2016.02.058 26967295

[B54] Shoda L. K. Woodhead J. L. Siler S. Q. Watkins P. B. Howell B. A. (2014). Linking physiology to toxicity using DILIsym(R), a mechanistic mathematical model of drug-induced liver injury. Biopharm. Drug Dispos. 35, 33–49. 10.1002/bdd.1878 24214486

[B55] Sidhu H. S. Srinivas R. Sadhotra A. (2017). Evaluate the effects of long-term valproic acid treatment on metabolic profiles in newly diagnosed or untreated female epileptic patients: a prospective study. Seizure 48, 15–21. 10.1016/j.seizure.2017.03.007 28365440

[B56] Siler S. Q. (2022). Applications of quantitative systems pharmacology (QSP) in drug development for NAFLD and NASH and its regulatory application. Pharm. Res. 39, 1789–1802. 10.1007/s11095-022-03295-x 35610402 PMC9314276

[B57] Silva M. F. Aires C. C. Luis P. B. Ruiter J. P. L I. J. Duran M. (2008). Valproic acid metabolism and its effects on mitochondrial fatty acid oxidation: a review. J. Inherit. Metab. Dis. 31, 205–216. 10.1007/s10545-008-0841-x 18392741

[B58] Tan Y. M. Worley R. R. Leonard J. A. Fisher J. W. (2018). Challenges associated with applying physiologically based pharmacokinetic modeling for public health decision-making. Toxicol. Sci. 162, 341–348. 10.1093/toxsci/kfy010 29385573 PMC6084449

[B59] Tiwari K. Kananathan S. Roberts M. G. Meyer J. P. Sharif Shohan M. U. Xavier A. (2021). Reproducibility in systems biology modelling. Mol. Syst. Biol. 17, e9982. 10.15252/msb.20209982 33620773 PMC7901289

[B60] Todisco S. Santarsiero A. Convertini P. DE Stefano G. Gilio M. Iacobazzi V. (2022). PPAR alpha as a metabolic modulator of the liver: role in the pathogenesis of nonalcoholic steatohepatitis (NASH). Biol. (Basel) 11, 792. 10.3390/biology11050792 35625520 PMC9138523

[B61] Tomson T. Battino D. Perucca E. (2016). Valproic acid after five decades of use in epilepsy: time to reconsider the indications of a time-honoured drug. Lancet Neurol. 15, 210–218. 10.1016/S1474-4422(15)00314-2 26655849

[B62] Varma M. V. Steyn S. J. Allerton C. EL-Kattan A. F. (2015). Predicting clearance mechanism in drug discovery: Extended clearance classification system (ECCS). Pharm. Res. 32, 3785–3802. 10.1007/s11095-015-1749-4 26155985

[B63] Xu S. Chen Y. Ma Y. Liu T. Zhao M. Wang Z. (2019). Lipidomic profiling reveals disruption of lipid metabolism in valproic acid-induced hepatotoxicity. Front. Pharmacol. 10, 819. 10.3389/fphar.2019.00819 31379584 PMC6659130

[B64] Yamashita H. Takenoshita M. Sakurai M. Bruick R. K. Henzel W. J. Shillinglaw W. (2001). A glucose-responsive transcription factor that regulates carbohydrate metabolism in the liver. Proc. Natl. Acad. Sci. U. S. A. 98, 9116–9121. 10.1073/pnas.161284298 11470916 PMC55382

[B65] Zaccara G. Messori A. Moroni F. (1988). Clinical pharmacokinetics of valproic acid--1988. Clin. Pharmacokinet. 15, 367–389. 10.2165/00003088-198815060-00002 3149565

