# Supplementary material for: Quantitative systems toxicology approach that integrates PBPK and core hepatic metabolism: a case study with valproic acid
Source: Front Pharmacol. 2026 Mar 20;17:1768190. doi: 10.3389/fphar.2026.1768190 (PMC13047117; doi:10.3389/fphar.2026.1768190)
Supplement: Supplementary file 1 [file DataSheet1.docx]

Supplementary Material

**
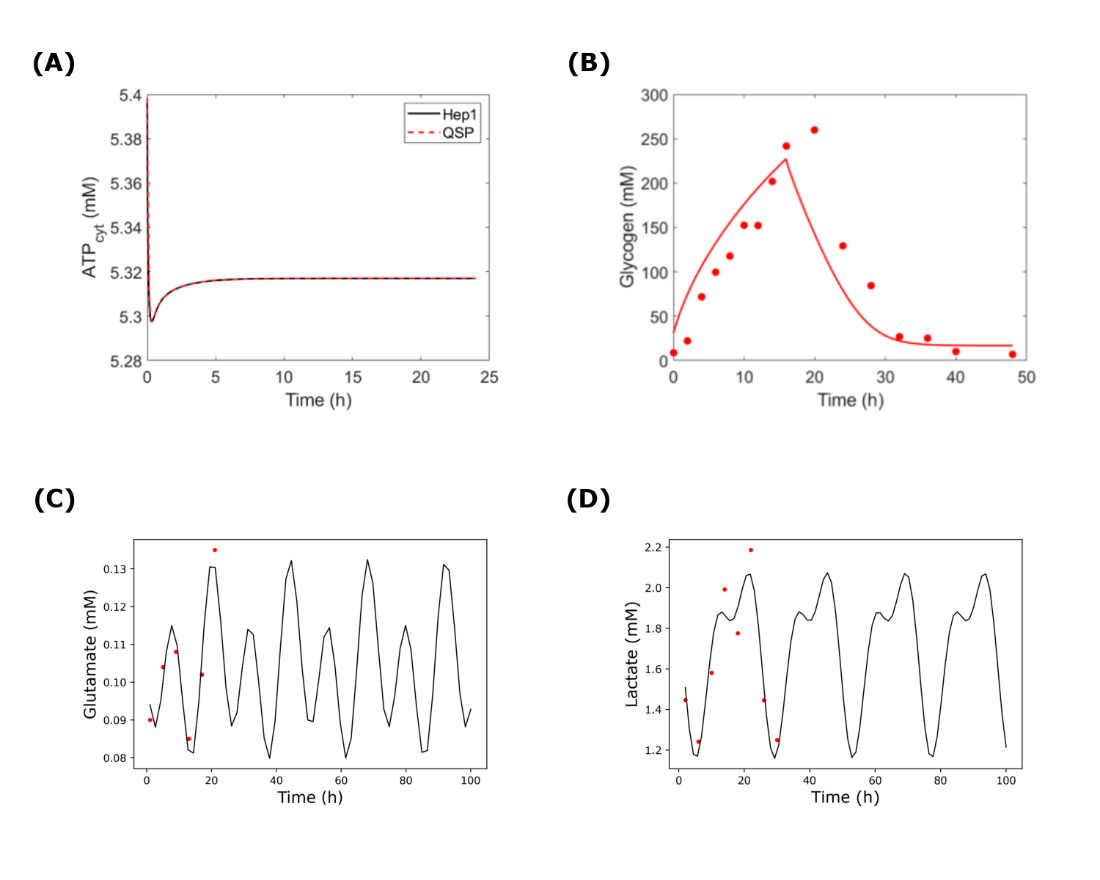
**

**Supplementary Figure S1. Verification of implemented HEPATOKIN1 model against the original model outcomes and experimental observations.** (**A**) Comparison of simulated cytosolic ATP concentrations (ATP_cyt_) using the original HEPATOKIN1 model (Berndt et al., 2018) (solid black line) with implemented HEPATOKIN1 model in Simcyp Designer (QSP, dotted red line). Reproduction of profiles of selected intermediates: (**B**) glycogen during fed (glucose = 8 mM) and fast (glucose = 4 mM) state, (**C**) diurnal profile of glutamate and (**D**) diurnal profile of lactate using experimental data (filled red circle) as reported in the original HEPATOKIN1 model.


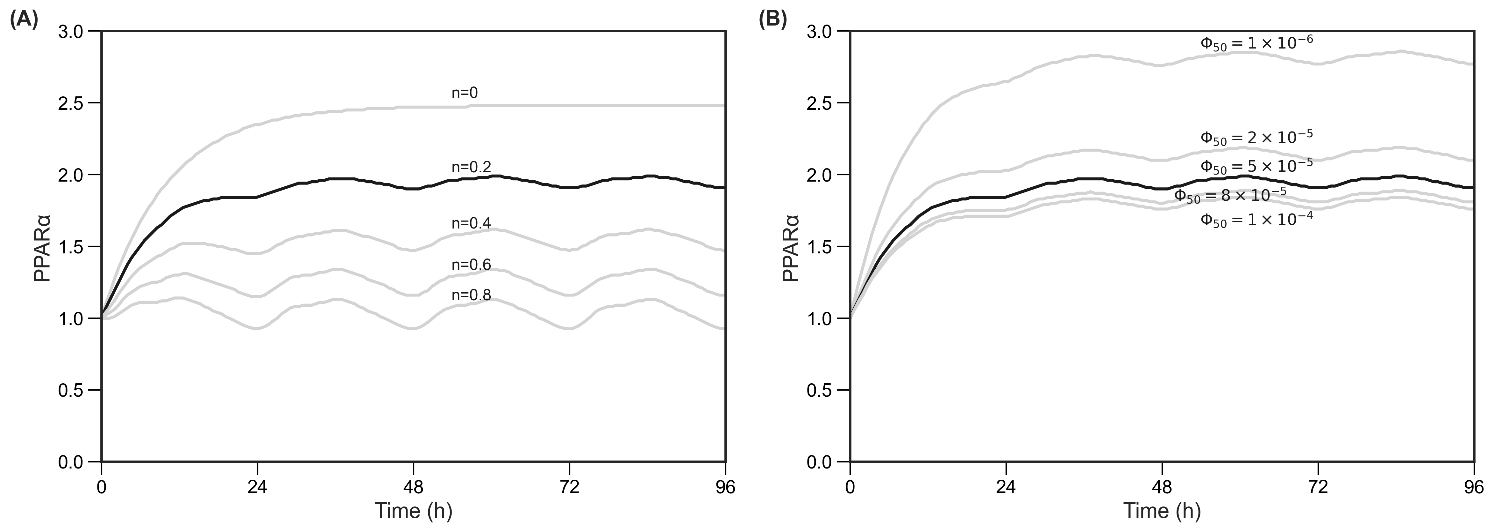


**Supplementary Figure S2. Sensitivity analysis of arbitrarily chosen parameters for transcription factor PPARα dynamics**. Changes in PPARα dynamics after changing values for (**A**) Hill’s coefficient (n) and (**B**) half-maximal cytosolic palmitate concentrations in mM (Ф_50_). The selected parameter value for the integrated model is indicated by the solid black line and non-selected parameter value outcomes are in solid grey lines. The ranges of Ф_50_ values used were guided from known palmitate concentrations from various VPA dosing simulations. PPARα, peroxisome proliferator-activated receptor α; VPA, valproic acid.


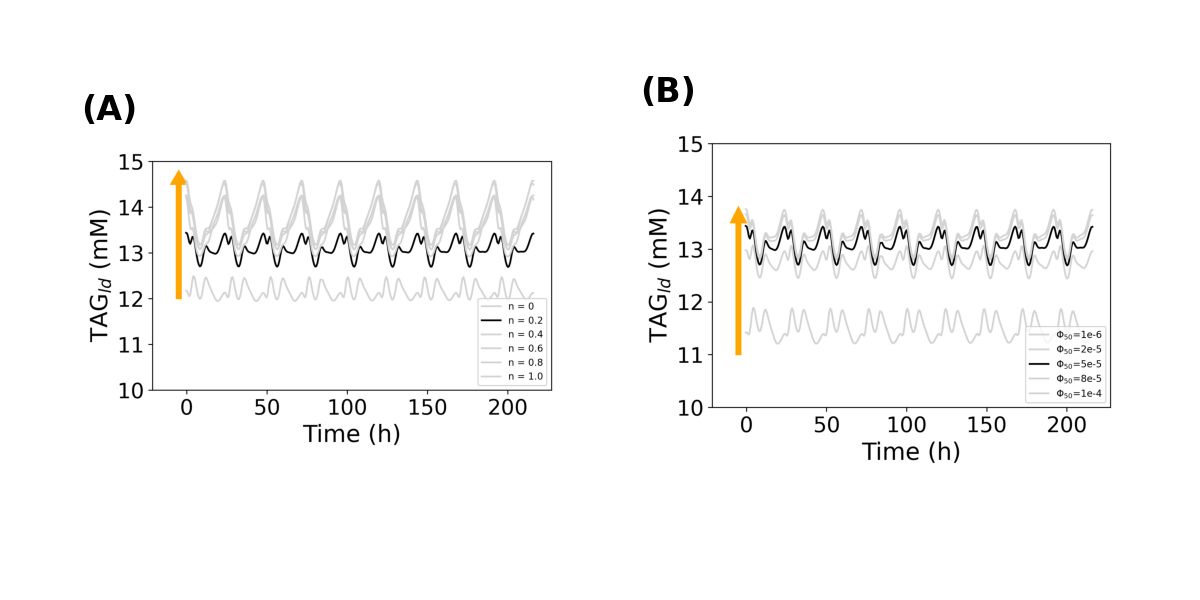


**Supplementary Figure S3. Sensitivity analysis of arbitrarily chosen parameters for transcription factor PPARα dynamics on simulated hepatic lipid droplet triglycerides (**$TAG_{ld}$**).** Changes in $TAG_{ld}$ concentrations after varying parameter values for PPARα dynamics: (A) Hill’s coefficient (n) and (B) half-maximal cytosolic palmitate concentrations in mM (Ф_50_). The selected parameter value for the integrated model is indicated by the solid black line and non-selected parameter value outcomes are in solid grey lines. The orange arrow in (A) denotes increasing simulated Hill coefficients bottom to top (n = 0 to n=1.0) and the arrow in (B) denotes increasing simulated Ф_50_ bottom to top (Ф_50_ = 1e-6 to Ф_50_ = 1e-4).


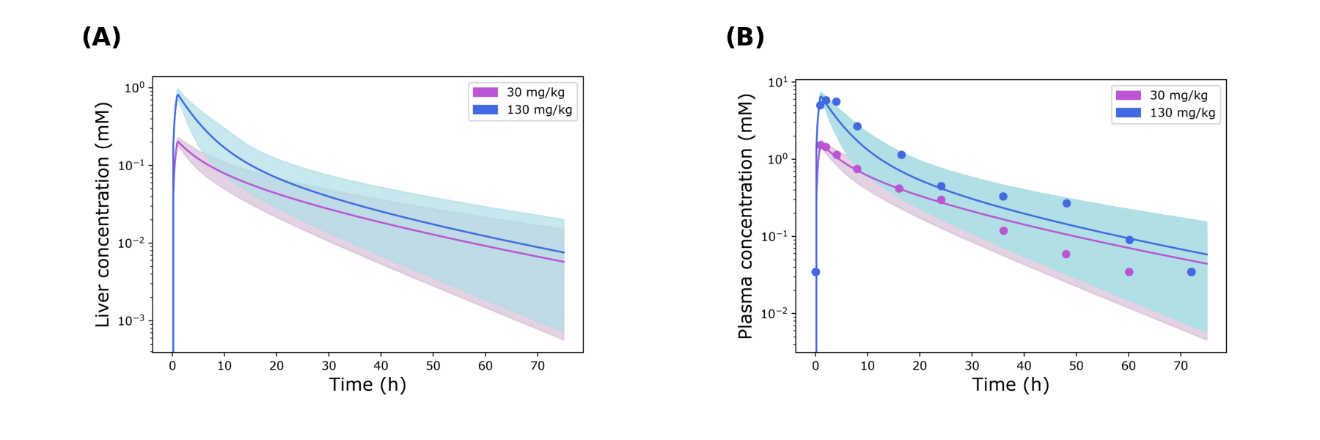


**Supplementary Figure S4. Simulated pharmacokinetic profiles of VPA.** Simulated total VPA concentrations in (**A**) liver and (**B**) plasma after single intravenous dose infused over 1 hour of 30 mg/kg and 130 mg/kg. Solid lines are simulated means and filled areas are simulated 95% confidence intervals. Data points are clinical observations (Georgoff et al., 2018). Simulation outcomes are from a virtual population of healthy volunteers (Sim-Healthy, n=8 for 10 trials) with 0.08 proportion of females (Supplementary Table S3). VPA, valproic acid.


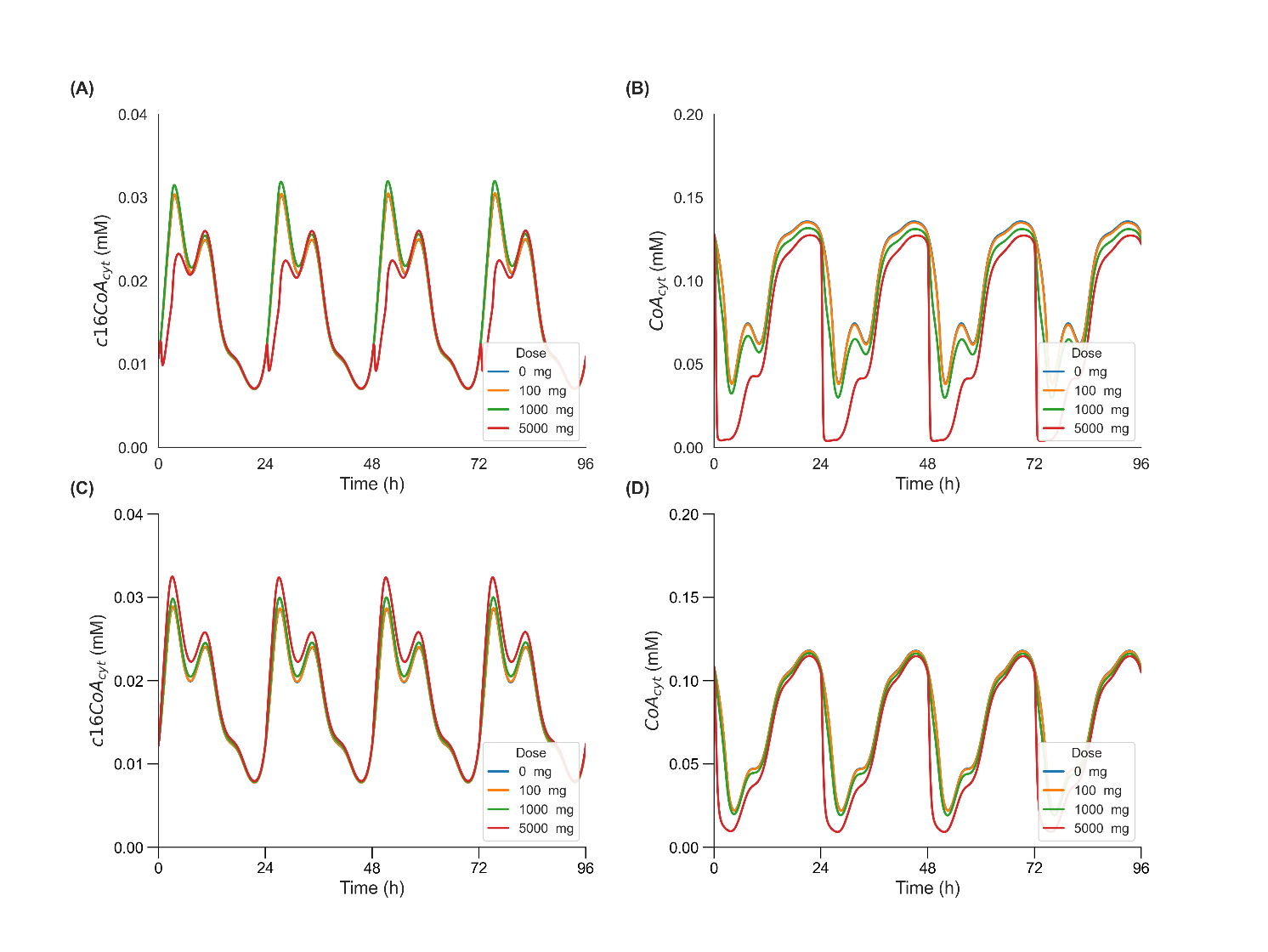


**Supplementary Figure S5. Effects of transcription factor PPARα regulation on VPA induced changes in cofactors of triacylglyceride.** Time-course profiles for concentrations of (**A**) cytosolic long chain acyl coenzyme A (C16CoA_cyt_) and (**B**) cytosolic coenzyme A (CoA_cyt_) without PPARα regulation, compared to (**C**) C16CoA_cyt_ and (**D**) CoA_cyt_ with PPARα regulation. Effects of once daily oral VPA dosing of 0, 100, 1000, and 5000 mg simulated in a single population representative virtual individuall . PPARα, peroxisome proliferator-activated receptor α; VPA, valproic acid.

**Supplementary Table S1.** Input parameters for VPA in the Simcyp Simulator. f_u_, fraction of drug unbound in plasma; pKa, acid dissociation constant; P_o:w_, octanol:water partition coefficient.

|  | **Parameter** | **Value /**  **Mean Value (CV%)** | **Source** |
| --- | --- | --- | --- |
| **Physicochemical properties** | Molecular weight (g/mol) | 144.21 | PubChem |
|  | LogP_o:w_ | 2.75 | PubChem measured from Sangster et al. (1993) |
|  | Compound type | Monoprotic acid |  |
|  | pKa | 4.80 | PubChem |
|  | Blood to plasma ratio, BP | 0.55 | (Ogungbenro et al., 2014) |
|  | Human serum albumin K_D_ (µM) | 107.53 | (Baker, 2010) |
|  | Human serum albumin binding $\gamma P_{T}$ (g/L) | 47.76 (0.1) | Simcyp Simulator Sim-Healthy virtual population (Curry et al., 2024) |
|  | Concentration dependent f_u_ | Enabled, Simcyp calcluated | Drug label for VPA (FDA, 2016) |
|  | Absorption model | First order absorption model |  |
| **Absorption** | First order absorption rate (ka)^*^ (1/h) | 2.52 | Simcyp predicted (V23r2); (Dutta and Reed, 2007) |
|  | Fraction absorbed (fa) | 0.99 | Simcyp predicted (V23r2) |
|  | Fraction unbound in enterocytes f_uGut_ | 1 | Assumed |
|  | Nominal flow in gut model Q_gut_ (L/h) | 16.00 | Simcyp predicted (V23r2) |
|  | Human jejunum effective permeability P_eff,man_ (10^-4^ cm/s) | 5.83 | Simcyp predicted using PSA and HBD (V23r2) |
|  | Polar surface area (Å^2^) | 37.30 | PubChem |
|  | Hydrogen bond donors | 1 | PubChem |
|  | Distribution model | Minimal PBPK model |  |
| **Distribution** | Volume of distribution at steady state V_ss_ (L/kg) | 0.13 | Predicted, method 2 (Rodgers and Rowland, 2007) (V23r2) |
|  | Hepatic portal vein blood flow rate Q_PV_  (% cardiac output) | 19 (Male)  21.5 (Female) | Simcyp Simulator Sim-Healthy virtual population (Curry et al., 2024) |
|  | Hepatic artery blood flow rate Q_HA_/Q_H_  (% cardiac output) | 6.5 (Male)  6.5 (Female) |  |
| **Single Adjusting Compartment (SAC)** | Rate of inflow to SAC K_in_ (1/h) | 0 | Not applied |
|  | Rate of outflow from SAC K_out_ (1/h) | 0 |  |
| **Elimination** | Elimination model | Whole organ metabolic clearance |  |
|  | *In vitro* hepatocyte intrinsic clearance (µL/min/10^6^ cells) | 0.22 | (Sylvia E. Escher, 2020) |
|  | Hepatocyte binding (f_u_ hepatocytes) | 0.95 |  |
|  | Typical renal clearance (mL/min) | 0 |  |

*VPA is considered an exemplar Extended Clearance Classification System (ECCS) class 1A compound with high permeability, ~ 90% contribution of metabolic hepatic clearance to systemic clearance, and no significant contribution of hepatic uptake or efflux transporters (Varma et al., 2015). In oral dosing, VPA demonstrates high bioavailability (F~ 1) that reflects high fraction absorbed (fa ~ 1), negligible first pass metabolism in the gut (Fg~ 1) and low hepatic first-pass metabolism. This is consistent with the low intrinsic hepatic clearance validated in intravenous dosing and without specialized release formulations VPA shows rapid first-order absorption with oral dosing (Dutta and Reed, 2007).

**Supplementary Table S2. List of enzymes regulated by PPARα.** Arrows indicate upregulation or downregulation of enzyme levels by peroxisome proliferator-activated receptor α (PPARα).

| **Enzyme** | **Name** | **Regulation** | **PPARα scaling** | **Source** |
| --- | --- | --- | --- | --- |
| AAT | Alanine amino transferase | ↑ | PPARα $\times$ AAT flux | (Kersten, 2014) |
| ACC1  ACC2 | Acetyl-CoA carboxylase | ↓ | 1/ PPARα $\times$ ACC1 flux  1/ PPARα $\times$ ACC2 flux | (Todisco et al., 2022) (Maldonado et al., 2018) |
| ACSL1  ACSL4  ACSL5 | Long chain acyl- CoA synthetase | ↑ | PPARα $\times$ ACSL1 flux  PPARα $\times$ ACSL4 flux  PPARα $\times$ ACSL5 flux | (Kersten, 2014) |
| ALDDH_gra_  ALDDHI  ALDDHII | Aldehyde dehydrogenase | ↑ | PPARα $\times$ ALDDH_gra_ flux  PPARα $\times$ ALDDHI flux  PPARα $\times$ ALDDHII flux | (Andrejeva et al., 2018) |
| ALDR | Aldehyde reductase | ↑ | PPARα $\times$ ALDR flux | (Maldonado et al., 2018) |
| ASL | Argininosuccinate lyase | ↑ | PPARα $\times$ ASL flux | (Maldonado et al., 2018) |
| C6CoA_MCDH_  C8CoA_MCDH_  C10CoA_MCDH_  C12CoA_MCDH_ | Medium chain acyl- CoA dehydrogenase | ↑ | PPARα $\times$ C6CoA_MCDH_ flux  PPARα $\times$ C8CoA_MCDH_ flux  PPARα $\times$ C10CoA_MCDH_ flux  PPARα $\times$ C12CoA_MCDH_ flux | (Maldonado et al., 2018),  (Desvergne and Wahli, 1999) |
| CACT | Carnitin-acylcarnitin translocase | ↑ | PPARα $\times$ CACT flux | (Gutgesell et al., 2009) |
| CPS | Carbamoyl-phosphate synthase | ↑ | PPARα $\times$ CPS flux | (Maldonado et al., 2018) |
| CPT1 | Carnitinpalmitoyl transferase 1 | ↑ | PPARα $\times$ CPT1 flux | (Desvergne and Wahli, 1999),  (Kersten, 2014) |
| FAS_C4_  FAS_C6_  FAS_C8_  FAS_C10_  FAS_C12_  FAS_C14_  FAS_C16_ | Fatty acid synthase | ↓ | 1/ PPARα $\times$ FAS_C4_ flux  1/ PPARα $\times$ FAS_C6_ flux  1/ PPARα $\times$ FAS_C8_flux  1/ PPARα $\times$ FAS_C10_ flux  1/ PPARα $\times$ FAS_C12_ flux  1/ PPARα $\times$ FAS_C14_ flux  1/ PPARα $\times$ FAS_C16_ flux | (Todisco et al., 2022) |
| G6P_er_ | D-glucose-6-phosphate phosphatase | ↑ | PPARα $\times$ G6P_er_ flux | (Kersten, 2014) |
| GLNASE | Glutaminase | ↑ | PPARα $\times$ GLNASE flux | (Maldonado et al., 2018) |
| HMG_syn_cyt_  HMG_syn_ | HMG- CoA synthase | ↑ | PPARα $\times$ HMG_syn_cyt_ flux  PPARα $\times$ HMG_syn_ flux | (Todisco et al., 2022) |
| ME | NADP dependent malic enzyme | ↑ | PPARα $\times$ ME flux | (Desvergne and Wahli, 1999) |
| OTC | Orinithine transcarbamylase | ↑ | PPARα $\times$ OTC flux | (Maldonado et al., 2018) |

**Supplementary Table S3. Varied physiological parameters in the virtual population matching the clinical trial population from Georgoff et al. for VPA PBPK model validation.** Georgoff et al. recruited healthy male and female subjects aged 18 – 65 with Body Mass Index between 18 kg/m^2^ and 30kg/m^2^ in 8-person cohorts with an 8% female proportion (Georgoff et al., 2018). This volunteer population was virtually matched by a 10 x 8-person virtual population of distinct healthy individuals with an 8% female proportion in the Simcyp Simulator.

| **Variable** | **Mean (95% confidence interval)** |
| --- | --- |
| Age (years) | 30.17 (27.17 – 30.83) |
| Body weight (kg) | 80.19 (76.67 – 82.04) |
| Height (cm) | 175.15 (173.57 – 176.49) |
| BMI (kg/m^2^) | 26.17 (25.03 – 26.76) |
| Cardiac output (L/h) | 347.73 (338.14 – 353.90) |
| Liver weight (g) | 1734.37 (1661.77 – 1773.36) |
| Haematocrit (%) | 42.14 (41.40 – 42.70) |
| Human Serum Albumin (g/L) | 47.76 (46.52 – 48.58) |

# References

ANDREJEVA, D., KUGLER, J. M., NGUYEN, H. T., MALMENDAL, A., HOLM, M. L., TOFT, B. G., LOYA, A. C. & COHEN, S. M. 2018. Metabolic control of PPAR activity by aldehyde dehydrogenase regulates invasive cell behavior and predicts survival in hepatocellular and renal clear cell carcinoma. *BMC Cancer,* 18**,** 1180.

BAKER, M. 2010. *Kinetic Determinants of Hepatic Uptake.* University of Sheffield.

BERNDT, N., BULIK, S., WALLACH, I., WUNSCH, T., KONIG, M., STOCKMANN, M., MEIERHOFER, D. & HOLZHUTTER, H. G. 2018. HEPATOKIN1 is a biochemistry-based model of liver metabolism for applications in medicine and pharmacology. *Nat Commun,* 9**,** 2386.

CURRY, L., ALRUBIA, S., BOIS, F. Y., CLAYTON, R., EL-KHATEEB, E., JOHNSON, T. N., FAISAL, M., NEUHOFF, S., WRAGG, K. & ROSTAMI-HODJEGAN, A. 2024. A guide to developing population files for physiologically-based pharmacokinetic modeling in the Simcyp Simulator. *CPT Pharmacometrics Syst Pharmacol,* 13**,** 1429–1447.

DESVERGNE, B. & WAHLI, W. 1999. Peroxisome proliferator-activated receptors: nuclear control of metabolism. *Endocr Rev,* 20**,** 649–88.

DUTTA, S. & REED, R. C. 2007. Distinct absorption characteristics of oral formulations of valproic acid/divalproex available in the United States. *Epilepsy Res,* 73**,** 275–83.

FDA. 2016. *DEPAKENE (valproic acid) Label* [Online]. Available: <https://www.accessdata.fda.gov/drugsatfda_docs/label/2016/018081s065_018082s048lbl.pdf> [Accessed].

GEORGOFF, P. E., NIKOLIAN, V. C., BONHAM, T., PAI, M. P., TAFATIA, C., HALAWEISH, I., TO, K., WATCHAROTONE, K., PARAMESWARAN, A., LUO, R., SUN, D. & ALAM, H. B. 2018. Safety and Tolerability of Intravenous Valproic Acid in Healthy Subjects: A Phase I Dose-Escalation Trial. *Clin Pharmacokinet,* 57**,** 209–219.

GUTGESELL, A., WEN, G., KONIG, B., KOCH, A., SPIELMANN, J., STANGL, G. I., EDER, K. & RINGSEIS, R. 2009. Mouse carnitine-acylcarnitine translocase (CACT) is transcriptionally regulated by PPARalpha and PPARdelta in liver cells. *Biochim Biophys Acta,* 1790**,** 1206–16.

KERSTEN, S. 2014. Integrated physiology and systems biology of PPARalpha. *Mol Metab,* 3**,** 354–71.

MALDONADO, E. M., FISHER, C. P., MAZZATTI, D. J., BARBER, A. L., TINDALL, M. J., PLANT, N. J., KIERZEK, A. M. & MOORE, J. B. 2018. Multi-scale, whole-system models of liver metabolic adaptation to fat and sugar in non-alcoholic fatty liver disease. *NPJ Syst Biol Appl,* 4**,** 33.

OGUNGBENRO, K., AARONS, L., CRESIM & EPI, C. P. G. 2014. A physiologically based pharmacokinetic model for Valproic acid in adults and children. *Eur J Pharm Sci,* 63**,** 45–52.

RODGERS, T. & ROWLAND, M. 2007. Mechanistic approaches to volume of distribution predictions: understanding the processes. *Pharm Res,* 24**,** 918–33.

SYLVIA E. ESCHER, A. B., ALICE LIMONCIEL, PAUL JENNINGS, BARBARA M. A. VAN VUGT-LUSSENBURG, BART VAN DE BURG, ENRICO MOMBELLI, FREDERIC BOIS, ALEJANDRO AGUAYO OROZCO, OLIVIER THIERRY TABOUREAU, BARBARA ZDRAZIL,ULF NORINDER, RICHARD MACLENNAN, PAUL WALKER, DOMENICO GADALETA, EMILIO BENFENATI, THOMAS EXNER, ANTHONY LONG, NAZANIN GOLBAMAKI, ERNEST MURRAY,DAVID J PONTING, CIARÁN FISHER, IAIN GARDNER, ANDREW WHITE, RABEA GRAEPEL, NANETTE VRIJENHOEK, BOB VAN DE WATER 2020. CASE STUDY ON THE USE OF INTEGRATED APPROACHES TO TESTING AND ASSESSMENT FOR PREDICTION OF A 90 DAY REPEATED DOSE TOXICITY STUDY (OECD 408) FOR 2-ETHYLBUTYRIC ACID USING A READ-ACROSS APPROACH FROM OTHER BRANCHED CARBOXYLIC ACIDS. OECD.

TODISCO, S., SANTARSIERO, A., CONVERTINI, P., DE STEFANO, G., GILIO, M., IACOBAZZI, V. & INFANTINO, V. 2022. PPAR Alpha as a Metabolic Modulator of the Liver: Role in the Pathogenesis of Nonalcoholic Steatohepatitis (NASH). *Biology (Basel),* 11.

VARMA, M. V., STEYN, S. J., ALLERTON, C. & EL-KATTAN, A. F. 2015. Predicting Clearance Mechanism in Drug Discovery: Extended Clearance Classification System (ECCS). *Pharm Res,* 32**,** 3785–802.
